# Endoscopic vs. External Dacryocystorhinostomy in Granulomatosis with Polyangiitis: A Scoping Review of the Literature and Our Experience with Endoscopic Dacryocystorhinostomy

**DOI:** 10.3390/jpm15070278

**Published:** 2025-07-01

**Authors:** Nitish Kumar, Lisa A. Marks, Pedro Lança Gomes, Devyani Lal

**Affiliations:** 1 Department of Otorhinolaryngology, Mayo Clinic in Arizona, Phoenix, AZ 85054, USA; kumar.nitish@mayo.edu (N.K.);; 2 Division of Education, Department of Library Services, Mayo Clinic in Arizona, Scottsdale, AZ 85259, USA; marks.lisa@mayo.edu

**Keywords:** autoimmune vasculitis, dacryocystorhinostomy, epiphora, nasolacrimal duct obstruction, granulomatosis with polyangiitis

## Abstract

**Background/Objectives**: Although endoscopic dacryocystorhinostomy (DCR) has been widely accepted as the procedure of choice for nasolacrimal duct obstruction (NLDO) management due to most etiologies, concerns regarding the reactivation of disease and involvement of surrounding structures add to hesitation in its utilization for granulomatosis with polyangiitis (GPA) patients. No study has directly compared outcomes of external vs. endoscopic DCR in GPA patients. This information can be helpful for patient counselling and choosing a personalized surgical approach for the best results. **Methods**: A scoping review of the literature was performed in January 2024. The following databases were searched using a combination of MeSH (Medical Subject Headings) and keywords: Ovid MEDLINE, Ovid EMBASE, Scopus, and Web of Science. This scoping review is not registered. Medical records of two GPA patients who underwent endoscopic DCR at our center were reviewed. **Results**: The search yielded 96 articles; 15 articles met the inclusion criteria for a full review. Six studies with 22 procedures reported 100% success with endoscopic DCR. Nine studies with 122 procedures reported success in 88.5% of cases with external DCRs. Additional perioperative immunosuppression was recommended in patients with severe mucosal inflammation. The case series presents the disease course, details of surgery, and perioperative management in two GPA patients with NLDO who underwent endoscopic DCR successfully. **Conclusions**: Endoscopic DCR was associated with equivalent or better success rates and lower complications compared to external DCR in GPA patients. Ensuring disease remission state and appropriate immunomodulatory therapy can help prevent the proposed risk of endonasal disease reactivation with endoscopic DCR.

## 1. Introduction

Over time, dacryocystorhinostomy (DCR) has evolved from external to endoscopic techniques, driven by advancements in technology and surgical precision. While external DCR was initially favored for its direct access to the lacrimal sac, drawbacks such as scarring and prolonged recovery led to the rise of minimally invasive endoscopic approaches. Enhanced visualization allows for precise osteotomy creation, reduced tissue trauma, and improved post-surgical healing. As expertise grew, studies showed comparable or superior success rates for endoscopic DCR, making it a preferred technique for nasolacrimal duct obstruction (NLDO) management due to its efficiency, reduced complications, and favorable cosmetic outcomes [1].

Management of epiphora due to NLDO in patients with granulomatosis with polyangiitis (GPA) is troublesome. Studies performed in the late 20th century espoused medical management. The rationale against surgery was based on the high surgical failure rate [2] and the fear that mucosal trauma induced by surgical procedures may cause GPA to flare up, leading to complications such as delayed wound healing, cutaneous fistula, and postoperative epistaxis. Studies published over the last three decades demonstrated the safety and efficacy of DCR in the quiescent phase of GPA [3,4]. Early DCR surgeons favored external DCR, assuming this approach would accomplish a significant osteotomy, minimize nasal mucosal trauma, and have good surgical access. Endoscopic DCR was conversely thought to be associated with a higher risk of complications and failure due to the possibility of distorted nasal anatomy, increased manipulation of nasal mucosa, small osteotomy, and the chances of vasculitic inflammation spreading to the orbit [5,6].

With growing experience, endoscopic DCR surgeons have demonstrated favorable results with minimal complications for epiphora management in GPA patients [5,6,7,8,9,10]. Targeted endonasal osteotomy allows for a wide marsupialization of the lacrimal sac while minimizing trauma to adjacent areas, hence reducing postoperative inflammation, scarring, and external wound complications [5,6]. Additionally, endoscopic visualization also allows for aggressive post-surgical wound debridement with the removal of early scar tissue and granulations, thus facilitating the long-term success of the procedure.

Despite advancements, existing research on external versus endoscopic DCR for GPA remains fragmented, with limited studies exploring the scope of surgical outcomes. Understanding the strengths, limitations, and gaps in these techniques can inform clinical decision-making for ophthalmologists, ENT surgeons, and rheumatologists.

Thus, we conducted a scoping review to explore the available literature on external and endoscopic DCR in GPA patients, aiming to identify trends, gaps, and considerations that may guide future research and surgical decision-making. Additionally, we present a review of two cases of endoscopic DCR performed by the senior author (D.L.) in severely symptomatic GPA patients, contributing a practical perspective to the existing literature.

## 2. Materials and Methods

A scoping review of the English language literature was performed to map available evidence on the surgical management of NLDO in GPA patients. This scoping review is not registered. The search strategy was designed to identify studies reporting outcomes of endoscopic and external DCR for epiphora resolution. The search was conducted on 23 January 2024, and updated on 20 May 2025, in the following databases: Ovid MEDLINE 1946 to present, Ovid EMBASE 1988 to week 3 2024, Scopus 1970 to present, and Web of Science 1975 to present. A combination of MeSH (Medical Subject Headings) terms and keywords was used to complete the search. MeSH terms included: Lacrimal Apparatus Diseases, granulomatosis with polyangiitis, dacryocystorhinostomy, and endoscopy. Keywords included: epiphora, Wegener* granulomatosis, dacryocystorhinostomy, external dacryocystorhinostomy, endonasal dacryocystorhinostomy, dacryocystectomy, external DCR, and endonasal DCR. MeSH terms and keywords were combined using the Boolean operators “OR” and “AND”.

The abstract review was completed by N.K., and the selected abstracts were reviewed by the senior author (D.L.) and N.K. together. Studies were included if they reported outcomes of surgical management of NLDO in terms of resolution of epiphora and were in the English language. Review articles and studies not reporting surgical success rates were excluded. Extracted data included epiphora resolution, immunosuppressant therapy, follow-up duration, and complications. The reporting of this scoping review was conducted and reported in accordance with the Preferred Reporting Items for Systematic Review and Meta-Analysis statement extension for Scoping Reviews (PRISMA-ScR) guidelines.

In addition to the literature review, we examined medical records of two GPA patients who underwent endoscopic DCR at our tertiary care center. These cases provide additional insights into clinical outcomes, post-surgical complications, immunomodulatory therapy, and disease progression during follow-up.

## 3. Results

**Figure 1:**
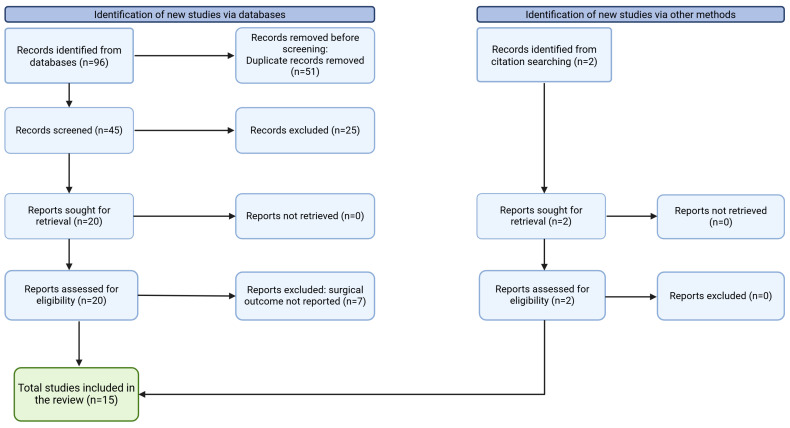
Preferred Reporting Items for Systematic Reviews and Meta-Analyses extension for Scoping Reviews (PRISMA-ScR) diagram.

The study selection process is outlined in the PRISMA-ScR flow diagram (Figure 1). The initial search identified 96 articles, with an additional two articles obtained through manual searching. After duplicate removal, 47 abstracts were screened, and 23 articles underwent full-text review. Ultimately, 15 studies met the inclusion criteria and were included in this scoping review. Among them, nine studies reported outcomes of external DCR, while six studies focused on endoscopic DCR (Table 1).

### 3.1. External Dacryocystorhinostomy

A total of 122 external DCR surgeries were reported, of which 108 surgeries (88.5%) resulted in a successful resolution of epiphora. These studies, published from 1987 [2] to 2019 [16], stated the use of external DCR as their choice of procedure for epiphora management due to NLDO in GPA.

The oldest published article by Jordan et al. in 1987 [2] advocated refraining from surgery in GPA patients due to the development of a nasocutaneous fistula at the external incision site after surgery. However, subsequent studies provided more optimistic findings, particularly when preoperative immunosuppressive therapy was optimized. Hardwig et al. [3] reported successful resolution of epiphora in 6 out of 10 external DCRs, with a follow-up ranging from 11 to 66 months, emphasizing the importance of GPA stabilization with immunosuppressive therapy before DCR. At about the same time, Glatt et al. [4] published two cases of successful external DCRs, highlighting the role of cyclophosphamide, azathioprine, and prednisone in preoperative management.

Later studies showed higher success rates, such as Kwan et al. [11] in 2000, reporting a 94% success rate in 15 external DCRs (14/15) performed in GPA patients, and advocated for additional immunosuppression in the perioperative period. Ghanem et al. [13] reported a case of GPA with dacryocystocele successfully managed with external DCR. Similarly, De Castro et al. [15] incorporated a modified Rain’s sinus stent placed into the lacrimal sac with external DCR, reporting complete resolution of epiphora in two patients, with no perioperative complications reported in the mean follow-up of 30 months. Other studies included one successful case report of bilateral DCRs [12], and two retrospective case reviews, with one reporting successful resolution of epiphora in 100% (18/18) of cases with a mean follow-up of 3.5 years [14], and the other reporting success in 91.4% (64/70) of cases with a mean follow-up of 2.5 years [16].

### 3.2. Endoscopic Dacryocystorhinostomy

A total of 22 endoscopic DCRs were reviewed for the management of epiphora in GPA patients, with a successful resolution in 100% of cases and no reported perioperative complications [5,6,7,8,9,10].

Wong et al. [7], for the first time in 1998, reported performing bilateral endoscopic DCRs for managing bilateral epiphora due to NLDO with a right-sided dacryocystocele, along with simultaneous revision endoscopic sinus surgery in a GPA patient. There was a successful resolution of epiphora during their follow-up of 5 months. Nine years later, Ishio et al. [8] reported one case of GPA who underwent endoscopic DCR with lacrimal stenting for NLDO and reported a successful outcome with a 16-month follow-up; although, epiphora recurred due to nasal mucosal inflammation at the DCR site, which required a short endoscopic procedure to restore patency of the nasolacrimal system. Eloy et al. [6] used a diode laser with endoscopic DCR, coupled with bicanalicular silicone intubation of the lacrimal system and silicone nasal splints for the lysis and prevention of intranasal adhesions in a GPA patient with extensive scarring in the nasal cavity with left-sided epiphora. Cannady et al. [9] conducted a retrospective review including seven GPA patients, reporting 100% surgical success over 50 months of follow-up. Morris et al. [5] reported five successful endoscopic DCRs in four GPA patients with a varied follow-up period of nine months to five years. The most recently published study was by Sweeney et al. [10], who retrospectively studied 51 endoscopic DCRs, with six cases of GPA. They advocated the use of high-volume budesonide nasal irrigations and serial endoscopic debridement to prevent crusting at the surgical site, and reported successful resolution of symptoms, including epiphora, in all cases.

### 3.3. Our Experience: Two Patients of GPA Who Underwent Endoscopic DCR

**Figure 2:**
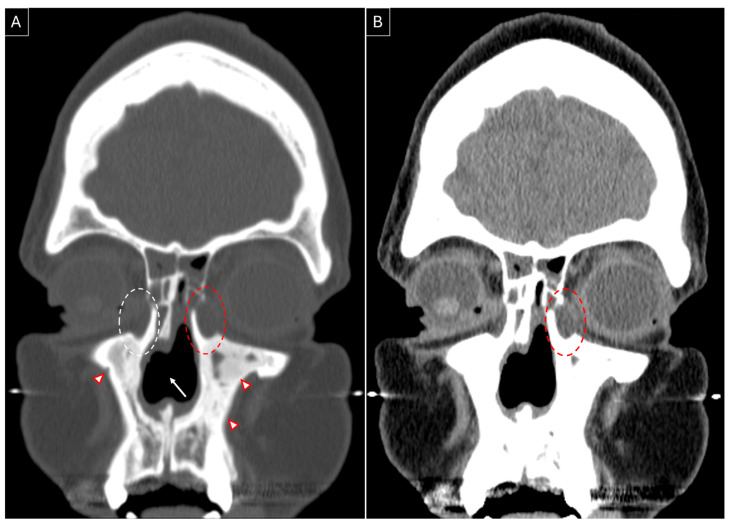
Non-contrasted sinus CT, coronal section, showing left-sided dacryocystocele (red dotted circle) in contrast to the right-sided lacrimal sac (white dotted circle). Presence of a nasal septal perforation (white arrow) and osteitic bone remodelling (red arrowheads) can be noted in both bone (**A**) and soft tissue (**B**) windows.

Case 1: A 48-year-old female patient presented in February 2021 with complaints of nasal congestion, drainage, facial pressure, and pain for 18 months. She had a poor response to medical management and developed loss of smell, altered taste sensation, left-sided epiphora, nasal bridge collapse, and diabetes insipidus, and underwent multiple failed endoscopic sinus surgeries. A solitary lesion in the lung was identified, and its biopsy was suggestive of GPA. She was prescribed rituximab with prednisone in December 2020. She was later diagnosed with subglottic stenosis and developed bilateral epiphora (left > right) in May 2021. She was stabilized on methotrexate, prednisone, and rituximab and tested negative for cytoplasmic anti-neutrophil cytoplasmic antibodies (cANCA). She underwent endoscopic DCR on her left side with the placement of Crawford stents in April 2022. Intraoperative findings included distorted anatomy, regression of bilateral middle turbinates, and formation of dacryocystocele (Figure 2). Stents were removed after three months. Granulation tissue formed at the site of DCR that needed repeated debridement to maintain patency of the stoma, which stabilized by the fifth postoperative month.

She underwent septal perforation repair in August 2022 but developed a smaller reperforation. She underwent external rhinoplasty with reconstruction of the bony pyramid and nasal valves with rib cartilage grafting in February 2023. She is still on regular follow-up with good disease control on low-dose weekly methotrexate.

Case 2: A 50-year-old female patient presented in November 2020 with a diagnosis of chronic rhinosinusitis, anti-phospholipid antibody syndrome, and suspected GPA. She had complaints of the gradual collapse of the nasal dorsum for the last 10 years, with intermittent nasal discharge and hoarseness of voice for 5 years. Perinuclear anti-neutrophil cytoplasmic antibodies (pANCA) were raised, due to which GPA was suspected. Improvement in voice was noted on 5 mg oral prednisone with 4 mg methotrexate weekly therapy. She underwent bilateral maxillary antrostomy, bilateral ethmoidectomy, and right frontal balloon sinuplasty in October 2019.

On her first visit, nasal endoscopy revealed a large subtotal septal perforation with extensive resorption of bilateral inferior turbinates, scanty crusting, extensive scarring in the bilateral maxillary ostium region, and a completely scarred right ostium. She underwent revision endoscopic sinus surgery with bilateral maxillary antrostomy, bilateral ethmoidectomy, and right hemi-modified Lothrop surgery with a Propel mini-stent placement. She also underwent direct laryngoscopy in the same setting with steroid injection in bilateral vocal cords for grade 3 sulcus vocalis and erythema over the vocal folds. She underwent open septorhinoplasty in April 2022, followed by major septal perforation repair.

**Figure 3:**
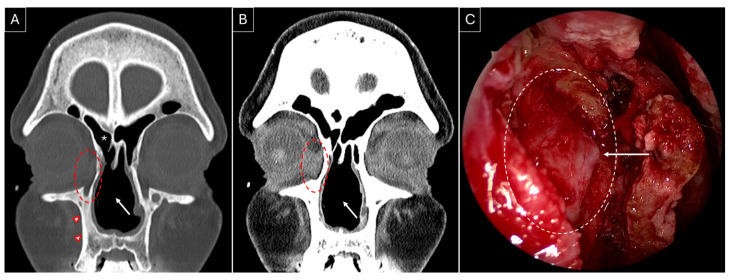
Non-contrasted sinus CT, coronal section, bone (**A**) and soft tissue (**B**) windows, showing right-sided dacryocystitis (red dotted circle). The presence of a nasal septal perforation (white arrow), osteitic bone remodelling (red arrowheads), and a previously performed right-sided hemi-modified Lothrop frontal sinusotomy can be noted (white asterisk). (**C**) Intraoperative endoscopic image of the right lacrimal sac medial wall (white dotted circle) after removal of the overlying lacrimal bone. The white arrow indicates tenting of the medial wall of the right lacrimal sac by the Bowman lacrimal probe.

She developed complaints of right-sided epiphora in April 2023 and underwent right-sided endoscopic DCR in August 2023; Figure 3A,B highlight the sinonasal radiological findings prior to surgery. Wide marsupialization of the lacrimal sac was ensured with the placement of a Crawford stent (Figure 3C). Histopathology revealed moderate inflammation, extensive fibrosis, and squamous metaplasia. Serial debridements were performed to deal with extensive crusting at the surgical site, which settled by the seventh postoperative month, along with a complete resolution of her symptoms with low-dose methotrexate maintenance therapy.

## 4. Discussion

Epiphora is observed in 7% of GPA patients [3] and is generally a late manifestation of the disease. The literature suggests a wide variability in the time interval between epiphora onset and GPA diagnosis, ranging from 3 months to 30 years [3,4,5,6,7,11,12,13], with a median interval of 8.4 years (interquartile range = 9). In our reviewed cases, one experienced GPA-related symptoms for 8 months, whereas the other had symptoms for 13 years before developing epiphora. Epiphora in GPA patients is attributed to NLDO due to extensive intranasal scarring and/or vasculitis of the lacrimal sac wall [13].

Surgical intervention during the active phase of GPA poses the risk of spreading inflammation to the surrounding skin, bone, and orbit, leading to the possibility of complications like nasocutaneous fistula and orbital manifestations like orbital myositis, keratopathy, and pseudotumor formation, which can even lead to compression optic neuropathy with an eventually possibility of blindness [17]. Other challenges, like increased intraoperative bleeding, poor visualization, increased surgical difficulty, and post-operative scarring, contribute to a considerable risk of epiphora recurrence. Therefore, surgery is only recommended once the disease achieves a quiescent stage, following appropriate immunosuppressive therapy [3]. Of the six studies discussing immunosuppressive therapy, prednisone was the most commonly used first-line agent [5,7,13,16], often supplemented with cyclophosphamide [4,5,7,13], azathioprine [4,5,7,16], or methotrexate [6,7]. Our first patient received methotrexate, prednisone, and rituximab during the intensive phase, and the other patient achieved stabilization with prednisone and methotrexate. Low-dose weekly methotrexate was used for maintenance therapy in both our cases.

Since the description of the contemporary endoscopic DCR technique in the year 2000 by Wormald [18], it gained widespread acceptance due to its advantages over external DCR, including no external scar or bruising, minimal damage to surrounding muscle with non-violation of the lacrimal pump mechanism, and faster recovery with minimal tissue trauma [19]. Despite these benefits, external DCR continued to be utilized in GPA patients as late as 2019 [16]. This could be due to the concerns that direct manipulation of the nasal mucosa and drilling of the nasal bone can lead to flaring up of the mucosal inflammation, bony necrosis, increased surgical difficulty, and a resulting smaller ostium, all leading to a higher risk of complications and recurrence [5]. However, recent evidence challenges this assumption, suggesting that minimal tissue dissection in endoscopic DCR compared to external DCR incites lesser inflammation post-operatively, reducing complications and recurrence risks [5]. The endoscopic approach also facilitates simultaneous sinonasal surgery, addressing coexisting disease in GPA patients.

A key observation from our review is the high success rate (100%) in endoscopic DCR over two decades (1998–2019), with no reported perioperative complications. While the sample size remains small (22 surgeries), several studies proposed methods to optimize surgical success in GPA patients, including the use of a preoperative CT for surgical planning [5,6,7,8,10], fiberoptic guidance and dye to accurately identify the lacrimal sac and distinguish the sac lumen from inflamed, thickened sac walls [8], use of silicone or Crawford tubes for lacrimal stenting to maintain postoperative patency [5,6,8,10], and high-volume postoperative saline and budesonide nasal irrigation, combined with early surgical debridement, to minimize scarring and obstruction [10]. However, none of these reports discuss patient-specific factors influencing the decision to use stenting. At our center, we routinely obtain preoperative CT scans for GPA patients undergoing endoscopic DCR, especially in cases with distorted intranasal anatomy to allow for intraoperative image guidance. It also aids in the assessment of coexisting sinus disease and the need for simultaneous sinus surgery. Our surgical protocol includes a posteriorly based mucoperiosteal flap for lacrimal bone exposure, which is then drilled to achieve complete sac exposure. A Bowman’s probe is inserted to tent the medial wall of the sac, guiding the incision (Figure 3C). Lacrimal puncta are serially dilated, followed by the use of Crawford tubes for lacrimal stenting, which are removed between 3 and 6 months postoperatively based on disease activity. Postoperative follow-up and serial endoscopic debridements are usually scheduled for the first week, sixth week, and third month. Patients are advised to perform low-pressure, high-volume saline nasal irrigations postoperatively. Given that most available data are limited to case reports and small case series (maximum sample size in reported case series = 7) [9], sharing our experience with two successful cases contributes valuable insights to the limited literature on endoscopic DCR outcomes in GPA-related epiphora.

In certain cases, external DCR may still be preferred, particularly in those with severely altered nasal anatomy where endoscopic access can be challenging, and can increase the risk of surrounding tissue injury and resultant orbital and sinonasal complications [6]. For a selected group of GPA patients with recurrent dacryocystitis with a history of multiple DCRs [6], extensive destruction of the DCR site [20], or in cases planned for external rhinoplasty where preservation of the nasal bone is of utmost importance [21], dacryocystectomy (DCT) is another surgical option. While DCT effectively eliminates infection, it does not address epiphora, making it a non-ideal option for patients prioritizing symptom relief [21]. These observations can be helpful for the treating physician to formulate and offer a personalized management plan to the GPA patient, offering a suitable choice of surgical management and appropriate immunomodulatory therapy guided by the different disease and anatomical factors associated with each patient to ensure the best possible outcome.

To ensure minimal tissue inflammation and reduce the perioperative complications, extra immunosuppression with the use of an increased dosage of the existing or addition of another agent to the immunosuppressive therapy is advised in the perioperative period [5,6,11,16,20]. This can be particularly useful for cases with higher preoperative mucosal inflammation.

This study represents the first scoping review comparing external vs. endoscopic DCR in GPA-related epiphora management, identifying knowledge gaps and methodological limitations. The key limitations of our review include the heterogeneous sample sizes of the two groups being compared (external DCR: 122 vs. endoscopic DCR: 22), increasing the risk of sampling bias. All the included studies were retrospective and of low-level evidence (Level 4: Case report or retrospective case review). Many studies report short-term surgical success, but long-term outcomes, including recurrence rates and mucosal healing, remain underrepresented. The severity of GPA at the time of surgery may affect results, but it is inconsistently reported, limiting the ability to assess its influence on surgical success rates. Variability in immunosuppressive regimens across patients and studies may also impact healing and recurrence rates, thus being an uncontrolled confounding factor in our study. The available data were inadequate to allow for a meta-analysis; therefore, this study remains observational in nature.

Although this review does not provide strong evidence regarding the selection of standard-of-care surgical approach for epiphora management in GPA patients, it highlights an important gap in current literature and underscores the need for future prospective trials to provide stronger clinical guidance. Additionally, emerging research should focus on optimizing surgical techniques by investigating the roles of intraoperative image guidance, use of optical fibers and dyes for precise localization of the lacrimal sac, patient-specific criteria for lacrimal stenting, and the comparative effectiveness of saline vs. corticosteroid nasal irrigation in GPA patients undergoing endoscopic DCR.

## 5. Conclusions

While endoscopic DCR is widely regarded as the preferred approach for NLDO management in most etiologies, GPA patients pose unique challenges. The continued utilization of external DCR, even two decades after the adoption of endoscopic DCR, suggests a lingering hesitation among surgeons in choosing endoscopic DCR as the standard of care. This review highlights the equivalent or superior outcomes with endoscopic DCR in GPA patients, coupled with minimal complications as compared to the external technique. The ability to perform simultaneous sinus surgery and easy postoperative debridement offers further advantages for optimized long-term outcomes.

A personalized surgical approach, considering individual patient and disease characteristics, is essential for ensuring the best possible outcomes. Larger, prospective studies comparing external vs. endoscopic techniques are needed to strengthen clinical recommendations and advocate for endoscopic DCR as the standard-of-care surgical approach in GPA-related epiphora management.

## Figures and Tables

**Table 1:** Summary of studies included in the scoping review.

Author Name	Year	No. of Surgeries for GPA Patients	Outcome	Follow-Up (Months)
External dacryocystorhinostomy				
Jordan, et al. [2]	1987	2	Wound necrosis, nasocutaneous fistula in both	
Glatt, et al. [4]	1990	2	Successful	8, 16
Hardwig, et al. [3]	1992	10	6 successful, recurrence in 4	11–66
Kwan, et al. [11]	2000	15	13 successful, 1 resolved after revision surgery, 1 failed	3–96
Knoch, et al. [12]	2003	2	Successful	108
Ghanem, et al. [13]	2004	1	Successful	12
Lee, et al. [14]	2012	18 (16 primary, 2 revision)	Successful	10–72
De Castro, et al. [15]	2013	2	Successful	30 (mean)
Stewart, et al. [16]	2019	70	64 successful, rest resolved after revision surgery	3–14
Endoscopic dacryocystorhinostomy				
Wong, et al. [7]	1998	2	Successful	5
Ishio, et al. [8]	2007	1	Successful, recurrence of symptoms resolved after short endonasal procedure	16
Eloy, et al. [6]	2009	1	Successful	6
Cannady, et al. [9]	2009	7	Successful	50 (mean)
Morris, et al. [5]	2010	5	Successful	9–60
Sweeney, et al. [10]	2018	6	Successful	Not reported

## Data Availability

The original contributions presented in this study are included in the article. Further inquiries can be directed to the corresponding author(s).

## Abbreviation

DCR	Dacryocystorhinostomy
NLDO	Nasolacrimal duct obstruction
GPA	Granulomatosis with polyangiitis
PRISMA-ScR	Preferred Reporting Items for Systematic Review and Meta-Analysis extension for Scoping Review
cANCA	Cytoplasmic antineutrophilic cytoplasmic antibodies
pANCA	Perinuclear antineutrophilic cytoplasmic antibodies
CT	Computed tomography
DCT	Dacryocystectomy
